# The Rights of Medical Experts as Witnesses

**Published:** 1868-12

**Authors:** 


The Rights of Medical Experts as Witnesses.—In the Police Court of Sacramento, during the trial of a case of alleged crime, a medical witness, Dr. Simmons, was called on by the defence to testify as to his knowledge of the facts of the case, which he did accordingly. His opinion was then demanded, as an expert, with regard to the mental condition of the defendant at the time of the alleged crime. The doctor answered very properly, that he had already declared all he knew in the case, but would decline giving an opinion as an expert until he was paid an honorable fee. The Court overruled the objection, and required his opinion, which he still declined to give; whereupon he was placed nominally under arrest for contempt, for the purpose of having the question settled by a higher court. It is desirable that physicians everywhere take the same course. It is enough for them to spend their time hanging about court-rooms from day to day as ordinary witnesses. This they will do as good citizens, great as the sacrifice often is. Beyond this, their professional knowledge, which forms their capital in business, is personal property; and courts have no more right to it than to their professional services in other directions, or to the professional services of lawyers, or the manual skill of mechanics. It is not to be inferred that physicians are wanting in liberality when they take this ground, or that the value of the fee is the main purpose. The question is one of principle, and may almost be put in this form: As physicians already spend three-fourths of their time in waiting on the poor and serving the public, without pay, has not this same generous public, through the courts, a legitimate claim on the remaining fraction of their time and services? That "one good turn deserves another," is not, we believe, a maxim of law, in its sinister sense, or in any sense. And yet the impression seems to prevail, that because the services of medical men can always be had, on call, for the poor, or in emergencies, without consideration of pay, therefore they have no reasonable claim for compensation under any circumstances, and should accept every dollar vouchsafed to them for their services with humility and thanksgiving. Justice to themselves and to each other, and regard for the younger members of the fraternity, require that this idea of the unmixed and unqualified philanthropy of the profession should be narrowed down in some degree—enough, at least, to let in dollars and cents for bread. We have little fear of an adverse decision in the case of Dr. Simmons. A similar case was lately decided in Chicago in favor of the physician. The habits and interests of lawyers would naturally lead them to a correct view of the question in the abstract. And it is only when he finds himself bound by his position to take a different view, or when he feels proud of his skill in carrying a wrong point, that a lawyer would be likely to oppose the claim of a medical expert for compensation.—*Pacific Medical and Surgical Journal.*

